# Pathophysiological Mapping of Experimental Heart Failure: Left and Right Ventricular Remodeling in Transverse Aortic Constriction Is Temporally, Kinetically and Structurally Distinct

**DOI:** 10.3389/fphys.2018.00472

**Published:** 2018-05-15

**Authors:** Mathew J. Platt, Jason S. Huber, Nadya Romanova, Keith R. Brunt, Jeremy A. Simpson

**Affiliations:** ^1^Department of Human Health & Nutritional Sciences, University of Guelph, Guelph, ON, Canada; ^2^IMPART Team Canada Investigator Network, Saint John, NB, Canada; ^3^Department of Pharmacology, Dalhousie Medicine New Brunswick, Saint John, NB, Canada

**Keywords:** diastolic dysfunction, pulmonary hypertension, left heart disease, concentric remodeling, mouse models

## Abstract

A growing proportion of heart failure (HF) patients present with impairments in both ventricles. Experimental pressure-overload (i.e., transverse aortic constriction, TAC) induces left ventricle (LV) hypertrophy and failure, as well as right ventricle (RV) dysfunction. However, little is known about the coordinated progression of biventricular dysfunction that occurs in TAC. Here we investigated the time course of systolic and diastolic function in both the LV and RV concurrently to improve our understanding of the chronology of events in TAC. Hemodynamic, histological, and morphometric assessments were obtained from the LV and RV at 2, 4, 9, and 18 weeks post-surgery.

**Results:** Systolic pressures peaked in both ventricles at 4 weeks, thereafter steadily declining in the LV, while remaining elevated in the RV. The LV and RV followed different structural and functional timelines, suggesting the patterns in one ventricle are independent from the opposing ventricle. RV hypertrophy/fibrosis and pulmonary arterial remodeling confirmed a progressive right-sided pathology. We further identified both compensation and decompensation in the LV with persistent concentric hypertrophy in both phases. Finally, diastolic impairments in both ventricles manifested as an intricate progression of multiple parameters that were not in agreement until overt systolic failure was evident.

**Conclusion:** We establish pulmonary hypertension was secondary to LV dysfunction, confirming TAC is a model of type II pulmonary hypertension. This study also challenges some common assumptions in experimental HF (e.g., the relationship between fibrosis and filling pressure) while addressing a knowledge gap with respect to temporality of RV remodeling in pressure-overload.

## Introduction

Heart failure (HF), a chronic condition characterized by the inability of the heart to meet the metabolic demands of the body, is an increasing epidemic and a leading cause of mortality in the developed world (Benjamin et al., 2017). As the general population continues to age, the prevalence of HF will increase (Heidenreich et al., 2011). HF can develop acutely (e.g., infarction, broken heart syndrome, drug overdose) or over years as a result of chronic stress (e.g., hypertension, atherosclerosis, diabetes, cardiomyopathy). While all HF is progressive, each cause has defining characteristics that manifest in their own time-frame for reasons that are not always expected or explainable. Unfortunately, the majority of clinical knowledge on this progression is either retrospective or derived from late-stage patients only after years of remodeling precipitate symptoms. Alternatively, animal models are used to investigate the chronology of cardiac events to a particular stress over time. Temporal studies in animal models provide insight into the time course of remodeling patterns (Mirsky et al., 1983; Inoko et al., 1994; Klotz et al., 2006), and are valuable in the search for disease mechanisms and treatment targets (Foster et al., 2017).

Transverse aortic constriction (TAC) was developed to investigate the left ventricle (LV) response to pressure-overload (Rockman et al., 1991) and is suggested as a model of pulmonary hypertension (Chen et al., 2012; Mohammed et al., 2012; van Nierop et al., 2013). From a clinical standpoint, there is a growing understanding that poor right ventricle (RV) function is an independent predictor of HF patient outcomes (Ghio et al., 2001; Karaye et al., 2010; Meyer et al., 2010). At the same time, an increasing proportion of patients with LV dysfunction are also being diagnosed with concurrent RV dysfunction (Zakeri and Mohammed, 2015; Rosenkranz et al., 2016). Experimental models like TAC recapitulate this ‘biventricular phenotype’ and are therefore of growing importance to pre-clinical investigations. In this study, we address a knowledge gap in the TAC model with respect to the chronology of hemodynamic, morphological and kinetic changes in the RV as compared to the LV. We hypothesize that pulmonary hypertension and the associated RV pathology will occur secondary to LV dysfunction (i.e., type II pulmonary hypertension), and that the patterns of remodeling will be distinct from those observed in the LV.

Diastolic dysfunction is growing concern requiring more research, given that it is more prevalent than systolic dysfunction in HF patients (Hogg et al., 2004), the general population (Fischer et al., 2003; Wan et al., 2014), and particularly in women (Ferreira et al., 2015). There are numerous parameters used to assess cardiac diastole, however, no studies have compared these different indices for their relative capacity to grade diastolic dysfunction in a model of HF over time. This makes comparisons between experimental models using different parameters difficult to interpret. In the present study, we asked the question: do invasive indices of diastolic function (i.e., dP/dt_min_, filling pressures, Tau) agree over time in TAC? We hypothesize that different indices of diastolic function will progress independently given the complexity of cardiac diastole and, likely, the different aspects captured by each parameter.

The salient findings in this study are: (1) Pulmonary hypertension was secondary to the increase in LV filling pressure and dysfunction, and RV and pulmonary vasculature remodeling was temporally distinct from the LV. (2) Determining the severity of diastolic dysfunction in either ventricle over time was complex, given that diastolic parameters were not in agreement until overt systolic dysfunction was apparent. And (3) the time course of structural and kinetic changes in both ventricles was interesting: interstitial fibrosis did not correlate with filling pressures in either ventricle, LV pressures did not predict RV pressures, and hypertrophy in the LV and RV had both early and late stage phenotypes that were not predictable from prevailing hemodynamics. Together, this study improves our understanding of the biventricular response to pressure-overload and firmly establishes TAC as a model of type II pulmonary hypertension.

## Materials and Methods

### Surgical Model

Briefly, 9-week-old male (32–40 g) CD-1 mice (Charles River Laboratory International Inc.), were anesthetized with an isoflurane/oxygen mix (2%:100%), intubated and connected to a ventilator (Harvard Apparatus). Mice were ventilated at 200 breaths per minute at 300 μL per breath. TAC was done as previously described (Allwood et al., 2014; Foster et al., 2017). Briefly, the 2nd and 3rd ribs were separated from their cartilaginous connections with the sternum to expose the aortic arch. The transverse aorta was isolated and constricted to 26-gauge blunted needle with 7-0 silk thread. Sham surgery was similar to TAC, absent only the placement of the 7-0 thread. All mice were housed on a 12-h light/dark cycle and with food and water *ad libitum*. This study was approved by the Animal Care Committee at the University of Guelph and all experiments were carried out in accordance with the guidelines from the Canadian Council on Animal Care.

### Echocardiographic Analysis

Mice were anesthetized with an isoflurane/oxygen mix (2%/100%). Echocardiography was performed using the Vevo2100 system (VisualSonics Inc., Toronto, ON, Canada) with the 40 MHz MS550D ultrasound transducer. Mice were kept at 37°C throughout data collection using a TH-5 rectal probe thermometer (Physiotemp Instruments LLC, Clifton, NJ, United States). Acquired M-mode images were analyzed with the LV-trace function from the cardiac package (VisualSonics Inc., Toronto, ON, Canada), and data was obtained over at least 5 heart beats as previously described (Platt et al., 2017). All measurements were made between noon and 5 pm.

### Hemodynamic Analysis

Mice were sacrificed at 2, 4, 9, or 18 weeks after TAC or sham surgery. Again, an isoflurane/oxygen mix (2%:100%) was used, and again mice were kept at 37°C while a 1.2F catheter (FTS-1211B-0018; Scisense Inc., London, ON, Canada) was inserted into the RV via the right jugular and into the LV via the right carotid. Hemodynamic signals were digitized at a sampling rate of 2 kHz and recorded using iWorx^®^ analytic software (Labscribe2, Dover, NH, United States). Data sets were analyzed with the removal of respiration artifacts. The presence of pulmonary hypertension was determined using the 99th percentile upper reference limit (i.e., greater than three standard deviations above the mean right ventricular systolic pressure in sham mice). Thus, 32.5 mmHg was deemed the physiological threshold for pulmonary hypertension in our model. Animals designated for morphometric analysis had organs removed, cleaned and weighed. Animals designated for histological analysis were exsanguinated and 10 mL of 1x PBS, 10 mL of 0.5 mol L^-1^ KCl, and 10 mL of 10% buffered formalin (VWR, Mississauga, ON, Canada) were perfused through the right carotid artery. Tissues were harvested, stored in 10% buffered formalin for 24 h, and then transferred to 70% ethanol until tissue processing.

### Histological Analysis

Cross sectional slices (∼5 μm) were obtained from the mid papillary region of the heart. Paraffin embedded sections were stained with either Picro-Sirius Red (PSR; 500 ml of saturated picric acid solution and 0.5 g of Direct Red 80 from Sigma Aldrich), to visualize interstitial fibrosis (staining cytoplasm yellow and collagen red), or Wheat Germ (Sigma Aldrich) to assess cardiomyocyte cross-sectional area (CSA) (staining cell borders green). Images were acquired using an Olympus FSX 100 light microscope and analyzed using Cell Sense software (Olympus, Tokyo, Japan). CSA was quantified only from cardiomyocytes with centralized nuclei. For analysis of lung tissue, fresh Verhoff Van Geison stain was prepared with reagents obtained from VWR (Mississauga, ON, Canada). This stains elastic fibers and nuclei purple/black, collagen fibers red and cytoplasm yellow. The medial thickness of muscularized arteries was expressed as a percentage of external medial diameter. Arteries with internal diameters < 35 μm were selected for quantifying the ratio of muscularized to non-muscularized arteries (Sheikh et al., 2014).

### Statistical Methods

All results are expressed as means ± SE unless otherwise indicated. Statistical analysis was performed using Prism (Graphpad Software Inc.). Statistical significance between sham and TAC mice at individual time-points was determined using the two-tailed Students *t*-test. A one-way ANOVA (Tukeys *post hoc*) was used to determine differences within one group over 18 weeks as all measures were terminal. Differences between sequential time-points were subsequently determined using a two-tailed Students *t*-test. Regression analyses were done with Prism 6.0. The threshold for significance was *p* < 0.05 in all cases unless otherwise specified.

## Results

### RV Dysfunction Develops Secondary to LV Dysfunction, With a Complex Progression of Diastolic Dysfunction in Both Ventricles

**FIGURE 1:**
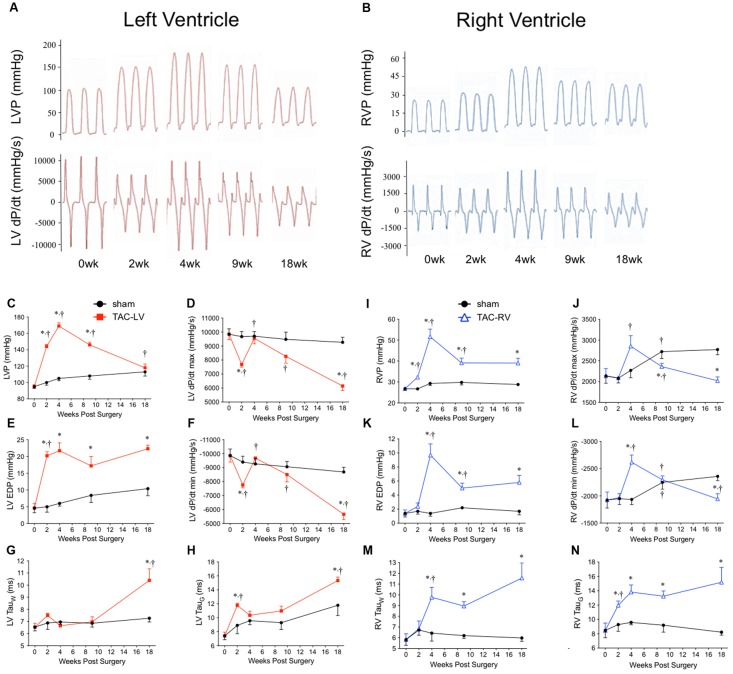
Hemodynamic characterization of the transverse aortic constriction (TAC) model in male CD1 mice over 18 weeks. Left panel (red) = left ventricular (LV) function. Right panel (blue) = right ventricular (RV) function. Representative hemodynamic tracings over 18 weeks from **(A)** the LV and **(B)** the RV. **(C)** Peak LV pressure (LVP), **(D)** LV dP/dt_max_, **(E)** left ventricular end diastolic pressure (LV EDP), **(F)** LV dP/dt_min_, **(G)** Tau (Weiss), and **(H)** Tau (Glantz) profile the temporal changes in LV hemodynamics over 18 weeks of TAC. **(I)** Peak RV pressure (RVP), **(J)** RV dP/dt_max_, **(K)** right ventricular end diastolic pressure (RV EDP), **(L)** RV dP/dt_min_, **(M)** Tau (Weiss), and **(N)** Tau (Glantz) profile the temporal changes in RV hemodynamics over 18 weeks of TAC. ^∗^*p* < 0.05 vs. age-matched sham. ^†^*p* < 0.05 vs. previous time point.

To account for changes in function as the mice aged, TAC’s were compared to age-matched surgical shams at all time-points. Representative tracings and a summary of invasive hemodynamic function over 18 weeks of TAC are presented in **Figure 1** and **Table 1** (for sham data, see **Table 2**).

**Table 1:** Invasive hemodynamics from the left and right ventricles in TAC.

		TAC			
		
Left Ventricle (LV)	Pre Surgery (*n* = 9)	2 weeks (*n* = 24)	4 weeks (*n* = 16)	9 weeks (*n* = 16)	18 weeks (*n* = 22)
LVP (mmHg)	95 ± 2	**144 ± 3**^∗,†^	**169 ± 4**^∗,†^	**146 ± 3**^∗,†^	118 ± 5^†^
EDP (mmHg)	4.6 ± 1.4	**20.2 ± 1.2**^∗,†^	**21.7 ± 2.4**^∗^	**17.2 ± 2.8**^∗^	**22.3 ± 1.1**^∗^
LV min Pressure (mmHg)	-2.8 ± 0.9	**9.0 ± 1.0**^∗,†^	**7.1 ± 1.6**^∗^	**7.5 ± 2.1**^∗^	**13.1 ± 1.2**^∗,†^
Systolic Pressure (mmHg)	88 ± 3	**142 ± 2**^∗,†^	**163 ± 5**^∗,†^	**144 ± 3**^∗,†^	118 ± 4^†^
Diastolic Pressure (mmHg)	61 ± 3	65 ± 2	**61 ± 3**^∗^	**57 ± 3**^∗^	**57 ± 3^∗^**
dP/dt_max_ (mmHg/s)	9838 ± 388	**7685 ± 191**^∗,†^	9552 ± 391^†^	8259 ± 486^†^	**6148 ± 332**^∗,†^
dP/dt@LVP40(mmHg/s)	9314 ± 401	**7116 ± 229**^∗,†^	8755 ± 441^†^	7512 ± 529	**5655 ± 274**^∗,†^
dP/dt_min_ (mmHg/s)	-9861 ± 464	**-7762 ± 205**^∗,†^	-9684 ± 464^†^	-8502 ± 519	**-5661 ± 373**^∗,†^
Tau (Weiss, ms)	6.54 ± 0.31	7.5 ± 0.18	6.65 ± 0.35^†^	6.97 ± 0.42	**10.39 ± 0.96**^∗,†^
Tau (Glantz, ms)	7.41 ± 0.55	**11.78 ± 0.30**^∗,†^	10.36 ± 0.57^†^	10.98 ± 0.70	**15.33 ± 0.51**^∗,†^
Tau (Logistics, ms)	8.27 ± 0.62	**13.46 ± 0.41**^∗,†^	11.98 ± 0.67	12.66 ± 0.84	**17.58 ± 0.58**^∗,†^
Tau (Mirsky, ms)	4.35 ± 0.19	**5.25 ± 0.08**^∗,†^	4.76 ± 0.17^†^	4.86 ± 0.17	6.04 ± 0.21^†^
HR (bpm)	573 ± 18	595 ± 9	599 ± 18	588 ± 12	574 ± 11
**Right Ventricle (RV)**	**Pre Surgery (*n* = 5)**	**2 weeks (*n* = 9)**	**4 weeks (*n* = 10)**	**9 weeks (*n* = 11)**	**18 weeks (*n* = 10)**
RVP (mmHg)	27 ± 1	**32 ± 1**^∗,†^	**52 ± 4**^∗,†^	**39 ± 2**^∗,†^	**39 ± 2**^∗^
EDP (mmHg)	1.4 ± 0.5	2.4 ± 0.5	**9.7 ± 1.6**^∗,†^	**5.0 ± 0.7**^∗,†^	**5.8 ± 1.0**^∗^
RV min Pressure (mmHg)	0.3 ± 0.3	0.5 ± 0.5	**6.2 ± 1.4**^∗,†^	1.8 ± 0.9^†^	**3.0 ± 0.8**^∗^
dP/dt_max_ (mmHg/s)	2138 ± 177	2088 ± 40	2853 ± 255^†^	**2362 ± 80**^∗,†^	**2021 ± 95**^∗,†^
dP/dt_min_ (mmHg/s)	-1923 ± 147	-1969 ± 73	**-2616 ± 132**^∗,†^	-2294 ± 72^†^	**-1944 ± 93**^∗,†^
Tau (Weiss, ms)	5.83 ± 0.54	6.84 ± 0.72	**9.77 ± 0.92**^∗,†^	**8.95 ± 0.41**^∗^	**11.56 ± 1.42**^∗^
Tau (Glantz, ms)	8.47 ± 1.03	**11.97 ± 0.59**^∗,†^	**13.81 ± 1.00**^∗^	**13.24 ± 0.73**^∗^	**15.18 ± 2.07**^∗^


*LVP, left ventricular pressure; EDP, end diastolic pressure; HR, heart rate; RVP, right ventricular pressure. Values are mean ± SEM. ^∗^*p* < 0.05 vs. age-matched sham. ^†^*p* < 0.05 vs. previous time point. Bolded numbers draw attention to significance vs. age-matched sham.*

**Table 2:** Invasive hemodynamics from the left and right ventricles in sham.

		sham			
		
Left Ventricle (LV)	Pre-surgery (*n* = 9)	2 weeks (*n* = 11)	4 weeks (*n* = 9)	9 weeks (*n* = 11)	18 weeks (*n* = 13)
LVP (mmHg)	97 ± 2	100 ± 3	103 ± 3	**106 ± 4**^∗^	**113 ± 5**^∗^
EDP (mmHg)	4.6 ± 1.4	5.0 ± 1.6	5.9 ± 0.8	**6.9 ± 2.1**^∗^	**9.9 ± 1.8**^∗^
LV min Pressure (mmHg)	-2.8 ± 0.9	-1.4 ± 0.9	-0.2 ± 1.0	0.2 ± 1.1	**1.2 ± 1.1**^∗^
Systolic Pressure (mmHg)	91 ± 3	95 ± 4	101 ± 4	100 ± 4	**110 ± 6**^∗^
Diastolic Pressure (mmHg)	64 ± 3	**72 ± 4**^∗,†^	**76 ± 3**^∗^	**75 ± 5**^∗^	**79 ± 4**^∗^
dP/dt_max_ (mmHg/s)	9838 ± 388	9671 ± 361	9703 ± 324	9478 ± 512	9325 ± 491
dP/dt@LVP40(mmHg/s)	9314 ± 401	9077 ± 364	8854 ± 259	8719 ± 474	8718 ± 461
dP/dt_min_ (mmHg/s)	-9861 ± 464	-9399 ± 412	-9266 ± 384	-9164 ± 376	**-8868 ± 334**^∗^
Tau (Weiss, ms)	6.37 ± 0.28	6.88 ± 0.53	6.96 ± 0.19	6.23 ± 0.30	7.17 ± 0.30
Tau (Glantz, ms)	7.54 ± 0.45	8.91 ± 1.19	**9.58 ± 0.58**^∗^	**9.06 ± 0.68**^∗^	**11.79 ± 1.48**^∗^
Tau (Logistics, ms)	8.27 ± 0.62	9.74 ± 1.39	**10.57 ± 0.64**^∗^	**10.03 ± 0.73**^∗^	**12.98 ± 1.61**^∗^
Tau (Mirsky, ms)	4.35 ± 0.19	4.7 ± 0.32	**5.17 ± 0.18**^∗^	4.91 ± 0.24	**5.59 ± 0.37**^∗^
HR (bpm)	573 ± 18	597 ± 15	590 ± 12	590 ± 13	568 ± 13
**Right Ventricle (RV)**	**Pre-surgery (*n* = 5)**	**2 weeks (*n* = 7)**	**4 weeks (*n* = 6)**	**9 weeks (*n* = 8)**	**18 weeks (*n* = 8)**
RVP (mmHg)	27 ± 1	28 ± 1	29 ± 1	**29 ± 1**^∗^	**30 ± 1**^∗^
EDP (mmHg)	1.4 ± 0.5	1.7 ± 0.4	2.4 ± 0.4	2.2 ± 0.2	2.1 ± 0.5
RV min Pressure (mmHg)	0.3 ± 0.3	0.3 ± 0.3	1.0 ± 0.2	0.7 ± 0.2	0.9 ± 0.1
dP/dt_max_ (mmHg/s)	2038 ± 177	2085 ± 115	2270 ± 176	**2722 ± 170**^∗,†^	**2845 ± 125**^∗^
dP/dt_min_ (mmHg/s)	-1923 ± 147	-1949 ± 107	-1934 ± 93	**-2247 ± 84**^∗,†^	**-2358 ± 77**^∗^
Tau (Weiss, ms)	5.83 ± 0.54	6.72 ± 0.49	6.43 ± 0.74	6.20 ± 0.26	5.98 ± 0.31
Tau (Glantz, ms)	8.47 ± 1.03	9.28 ± 0.95	9.58 ± 0.33	9.19 ± 0.96	8.52 ± 0.41


*LVP, left ventricular pressure; EDP, end diastolic pressure; HR, heart rate; RVP, right ventricle pressure. Values are mean ± SEM. ^∗^*p* < 0.05 vs. pre-surgery values. †*p* < 0.05 vs. previous time point. Bolded numbers draw attention to significance vs. pre-surgery values.*

LV pressure (LVP; **Figure 1C**) peaked at 4 weeks before progressively declining toward sham values by 18 weeks. RV pressure (RVP; **Figure 1I**) peaked at 4 weeks, but unlike the LVP, plateaued from 9 to 18 weeks. Of importance for the chronology of this model, pulmonary hypertension did not develop until 4 weeks. Only 11% (1/9) of TAC mice at 2 weeks had pulmonary hypertension. By 4 weeks, incidence of pulmonary hypertension rose to 90% (9/10) and was 75% thereafter (16/21).

To evaluate systolic function, the first positive derivative of ventricular pressure (dP/dt_max_) was used as an index of contractility (**Figures 1D,J**). LV dP/dt_max_ initially decreased at 2 weeks before rebounding by 4 weeks and progressively declining thereafter. In the RV, dP/dt_max_ was unchanged until increasing at 4 weeks before also declining thereafter.

To assess diastolic dysfunction, we used three different indices: the first negative derivative of ventricular pressure (dP/dt_min_), end diastolic pressure (EDP) and two methods to calculate the relaxation time constant (i.e., Tau Weiss; Tau_W_ and Tau Glantz; Tau_G_) (**Figures 1E–H,K–N** and **Table 1**). LV EDP increased ∼400% by 2 weeks and plateaued thereafter (**Figure 1E**). LV dP/dt_min_ followed the same trend as LV dP/dt_max_, initially reduced at 2 weeks, normalizing by 4 weeks and then progressively declining out to 18 weeks (**Figure 1F**). LV Tau_W_ increased only by 18 weeks (**Figure 1G**) while Tau_G_ was elevated both at 2 and 18 weeks (**Figure 1H**). In the RV, EDP initially increased by 4 weeks before partially returning toward sham levels at an elevated plateau between 9 and 18 weeks (**Figure 1K**). RV dP/dt_min_ followed the same pattern as RV dP/dt_max_, increasing at 4 weeks before declining out to 18 weeks (**Figure 1L**). RV Tau_G_ increased at 2 weeks (**Figure 1N**) while Tau_W_ increased at 4 weeks (**Figure 1M**); both stayed elevated from sham thereafter.

In sham mice, LV and RV pressure, as well as systolic and diastolic function also changed over 18 weeks. LVP increased by ∼20%, while RVP increased by ∼10% (**Figures 1C,I**). Interestingly, while LV dP/dt_max_ was stable (changing by <10% over 18 weeks), RV dP/dt_max_ increased ∼30% between 2 and 9 weeks (**Figures 1D,J**). In the LV, most measures of diastolic function declined by 18 weeks (EDP and Tau_G_ increased by ∼60%, while dP/dt_min_ declined by 10%), yet Tau_W_ remained unchanged (**Table 2**). Alternatively, in the RV, measures of diastolic function either improved (dP/dt_min_ increased by ∼40%) or were unchanged (EDP, Tau_G_ and Tau_W_ were stable; **Table 2**).

### Interstitial Fibrosis Did Not Correlate With Diastolic Function in Either Ventricle Over Time

**FIGURE 2:**
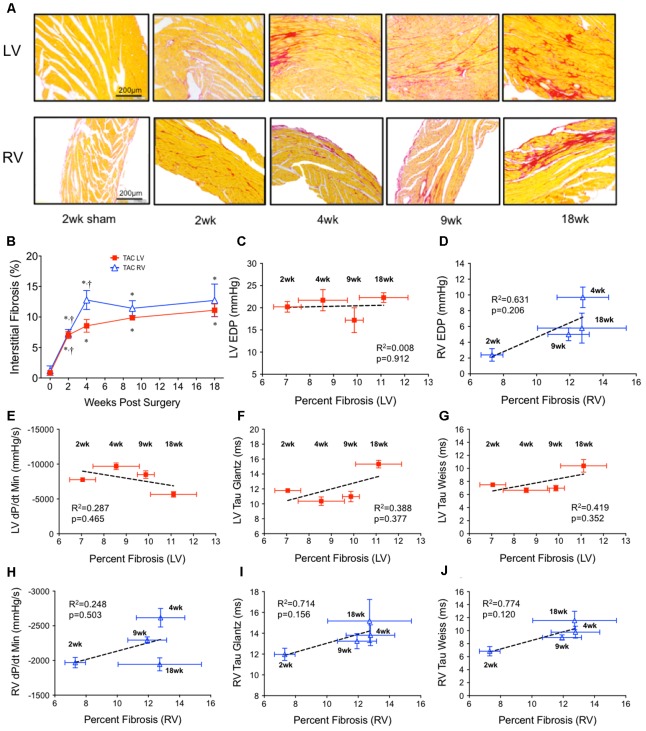
Ventricular interstitial fibrosis in both ventricles over 18 weeks of TAC. **(A)** Representative images stained with Picrosirius Red (top panel; left ventricle; LV, bottom panel; right ventricle; RV). **(B)** Average interstitial fibrosis over time in both ventricles (*n* = 6 for each point). Sham was unchanged over 18 weeks and was represented by the point at *x* = 0. ^∗^*p* < 0.05 vs. age-matched sham. ^†^*p* < 0.05 vs. previous time point. Linear regression was run on correlations between average fibrosis and all invasive indicies of diastolic function: **(C)** LV end diastolic pressure (LV EDP), **(D)** RV end diastolic pressure (RV EDP), **(E)** LV dP/dt_min_, **(F)** LV Tau Glantz, **(G)** LV Tau Weiss, **(H)** RV dP/dt_min_, **(I)** RV Tau Glantz, **(J)** RV Tau Weiss.

Elevations in EDP are sometimes reconciled as the product of fibrotic remodeling. To investigate this relationship over time in our model, histological sections of the ventricles were stained with PSR (**Figure 2A**). In the LV, interstitial fibrosis progressively increased over 18 weeks, initially increasing ∼8 fold by 2 weeks with a further 50% increase out to 18 weeks (**Figure 2B**). In the RV, interstitial fibrosis increased linearly ∼10 fold by 4 weeks, stabilizing thereafter (**Figure 2B**).

Regression did not find a significant non-zero relationship between EDP and fibrosis over time in either the LV (**Figure 2C**) or RV (**Figure 2D**). Of an interesting note was that interstitial fibrosis increased ∼500% in the RV by 2 weeks without a concurrent change in RV EDP.

To investigate if fibrosis correlated with any other parameter of diastolic function, we compared the relationship between fibrosis and dP/dt_min_, Tau_G_ and Tau_W_ in both the LV (**Figures 2E–G**) and RV (**Figures 2H–J**). No parameter consistently correlated over time. At best, in the RV only, there was a dichotomy between early and late fibrosis and diastolic dysfunction.

### Pressure-Overload Resulted in Divergent Patterns of Hypertrophy Between the LV and RV Over Time

**FIGURE 3:**
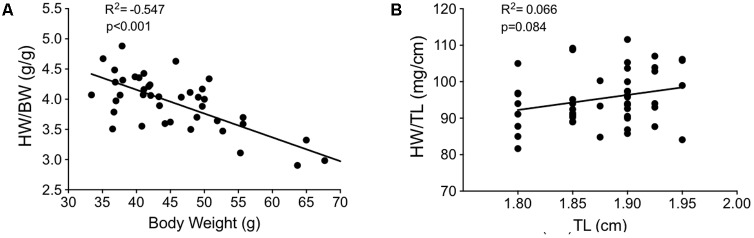
Correlation plots between measures of body growth and cardiac growth in sham mice between 9 and 27 weeks of age (*n* = 40). **(A)** Heart weight/body weight (HW/BW) plotted against body weight. **(B)** Heart weight/tibial length (HW/TL) plotted against tibial length. The lack of slope in **(B)** indicates HW/TL is less impacted by growth (i.e., changes in the denominator) than HW/BW. This suggests that normalizing cardiac weight to TL is a better method for determining cardiac hypertrophy between groups of varying weight/age.

**FIGURE 4:**
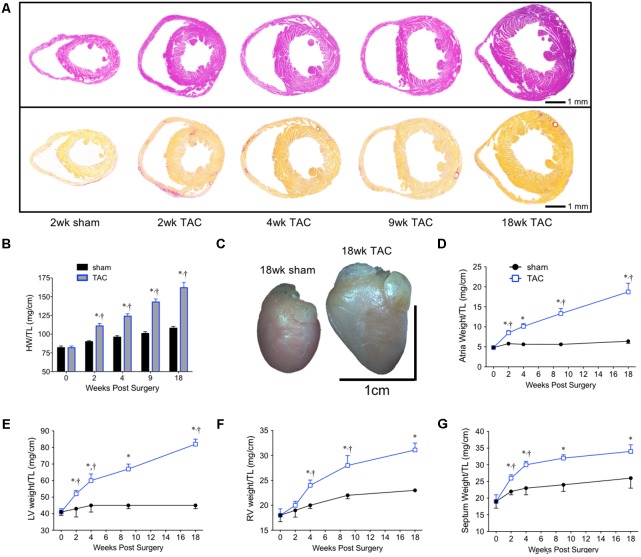
Summary of cardiac morphometrics following 18 weeks of TAC. **(A)** Representative cross-sectional images of the heart stained with H&E (top row) and PSR (bottom row). **(B)** Whole heart weight normalized to tibial length (HW/TL). **(C)** An 18 week sham (left) and TAC (right) heart. Normalized chamber-specific growth curves from the **(D)** atria, **(E)** left ventricle (LV), **(F)** right ventricle (RV), and **(G)** septum are presented. ^∗^*p* < 0.05 vs. age-matched sham. ^†^*p* < 0.05 vs. previous time point.

To investigate the hypertrophic response following LV pressure-overload, whole hearts (ventricles, septum and atria) were weighed and normalized to tibial length (TL). Weights were normalized to TL as increasing BW skewed the HW/BW ratio in sham mice, while HW/TL in the same mice was stable across normal growth (**Figure 3**). Representative H&E (top panel) and PSR (bottom panels) cross-sectional images of the heart are presented in **Figure 4A**. Whole heart hypertrophy increased steadily (**Figure 4B**), culminating in significantly larger hearts by 18 weeks of TAC (**Figure 4C**). To evaluate chamber-specific hypertrophy, atrial, LV, RV and septal weights were normalized to TL. The atrial/TL and LV/TL ratio increased steadily across all time points(**Figures 4D,E**). RV/TL did not increase until 4 weeks of TAC (**Figure 4F**) while the septum/TL ratio increased to 4 weeks where it plateaued and increased proportional to sham out to 18 weeks (**Figure 4G**). Expanded morphometrics are presented in **Table 3** (TAC) and **Table 4** (sham).

**Table 3:** Morphometric characteristics of TAC mice.

		TAC			
		
Variable	Pre-surgery (*n* = 11)	2 weeks (*n* = 16)	4 weeks (*n* = 16)	9 weeks (*n* = 15)	18 weeks (*n* = 14)
BW (g)	37.2 ± 0.7	**38.3 ± 2.9**^∗^	**40.8 ± 3.1**^∗,†^	**45.2 ± 7.2**^∗,†^	**44.1 ± 7.1**^∗^
TL (cm)	1.84 ± 0.02	1.84 ± 0.04	1.87 ± 0.03^†^	1.90 ± 0.03	1.93 ± 0.03^†^
Heart Weight (mg)	152 ± 4	**200 ± 24**^∗,†^	**235 ± 30**^∗,†^	**270 ± 40**^∗,†^	**312 ± 59**^∗,†^
Atria Weight (mg)	9 ± 1	**16 ± 6**^∗,†^	**20 ± 7**^∗,†^	**25 ± 12**^∗,†^	**36 ± 18**^∗,†^
LV Weight (mg)	79 ± 3	**98 ± 8**^∗,†^	**112 ± 24**^∗^	**128 ± 17**^∗^	**158 ± 16**^∗,†^
LV CSA (μm^2^)	189 ± 15	**296 ± 19**^∗,†^	**373 ± 44**^∗,†^	**471 ± 52**^∗,†^	**658 ± 87**^∗,†^
RV Weight (mg)	35 ± 2	36 ± 5	**45 ± 9**^∗,†^	**54 ± 13**^∗,†^	**59 ± 11**^∗^
RV CSA (μm^2^)	150 ± 14	168 ± 9	**234** ±**14**^∗^	**237 ± 12**^∗^	**241 ± 17**^∗^
Septum Weight (mg)	37 ± 3	**49 ± 7**^∗,†^	**57 ± 10**^∗,†^	**60 ± 5**^∗^	**66 ± 9**^∗^
Lung Wet Weight (mg)	221 ± 12	230 ± 49	273 ± 73^†^	**349 ± 152**^∗^	**450 ± 187**^∗^
Lung Wet/Dry Ratio	4.23 ± 0.09	4.26 ± 0.86	4.34 ± 0.58	4.30 ± 0.67	4.23 ± 0.94
Kidney Weight avg (mg)	269 ± 10	**269 ± 37**^∗^	**272 ± 39**^∗^	295 ± 42	316 ± 72
Spleen Weight (mg)	97 ± 6	**139 ± 27**^∗,†^	113 ± 15^†^	128 ± 28	**171 ± 35**^∗,†^
Liver Weight (g)	1.68 ± 0.14	1.87 ± 0.13	1.89 ± 0.32	2.08 ± 0.44	**2.07 ± 0.24**^∗^


*BW, body weight; TL, tibial length; LV, left ventricle; RV, right ventricle; Values are mean ± SD. ^∗^*p* < 0.05 vs. age-matched sham. ^†^*p* < 0.05 vs. previous time point. Bolded numbers draw attention to significance vs. age-matched sham.*

**Table 4:** Morphometric characteristics of sham mice.

		sham			
		
Variable	Pre-surgery (*n* = 11)	2 weeks (*n* = 8)	4 weeks (*n* = 10)	9 weeks (*n* = 9)	18 weeks (*n* = 10)
BW (g)	37.2 ± 2.3	**40.4 ± 3.1**^∗,†^	**44.0 ± 4.2**^∗,†^	**51.0 ± 7.0**^∗,†^	**55.1 ± 10.0**^∗,†^
TL (cm)	1.84 ± 0.05	1.85 ± 0.03	**1.88 ± 0.03**^∗,†^	**1.91 ± 0.02**^∗,†^	**1.92 ± 0.04**^∗^
Heart Weight (mg)	158 ± 14	**171 ± 10**^∗,†^	**181 ± 9**^∗^	**191 ± 14**^∗,†^	**200 ± 16**^∗^
Atria Weight (mg)	9 ± 2	10 ± 2	11 ± 1	11 ± 1	**12 ± 3**^∗^
LV Weight (mg)	79 ± 7	83 ± 10	85 ± 9	88 ± 8	**91 ± 3**^∗^
LV CSA (μm^2^) (n = 4)	189 ± 15	191 ± 11	196 ± 13	209 ± 12	213 ± 13
RV Weight (mg)	33 ± 5	37 ± 6	**41 ± 2**^∗^	**44 ± 3**^∗^	**45 ± 2**^∗^
RV CSA (μm^2^) (n = 4)	150 ± 9	154 ± 10	160 ± 11	166 ± 10	171 ± 12
Septum Weight (mg)	37 ± 6	41 ± 7	44 ± 8	**48 ± 8**^∗^	**52 ± 7**^∗^
Lung Wet Weight (mg)	221 ± 28	235 ± 32	252 ± 17	238 ± 44	235 ± 48
Lung Wet/Dry Ratio	4.23 ± 0.23	4.25 ± 0.71	4.51 ± 0.16	4.30 ± 0.42	4.49 ± 0.70
Kidney Weight avg (mg)	269 ± 24	**305 ± 23**^∗,†^	**318 ± 18**^∗^	**335 ± 30**^∗^	**342 ± 31**^∗^
Spleen Weight (mg)	97 ± 14	**112 ± 16**^∗,†^	107 ± 15	**115 ± 9**^∗^	**133 ± 7**^∗,†^
Liver Weight (g)	1.68 ± 0.34	1.99 ± 0.16	**2.10 ± 0.29**^∗^	**2.24 ± 0.36**^∗^	**3.01 ± 0.65**^∗,†^


*BW, body weight; TL, tibial length; LV, left ventricle; RV, right ventricle; Values are mean ± SD. ^∗^*p* < 0.05 vs. pre-surgery values. ^†^*p* < 0.05 vs. previous time point. Bolded numbers draw attention to significance vs. pre-surgery values.*

**FIGURE 5:**
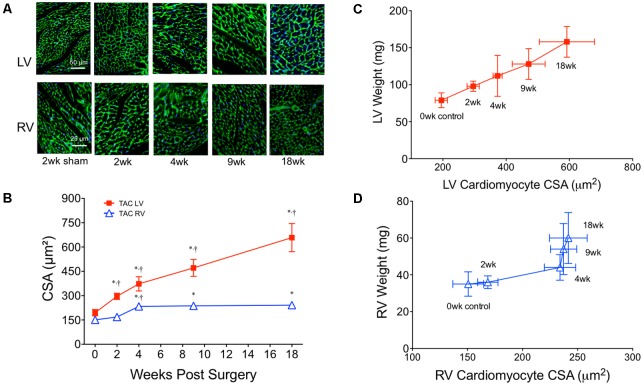
Cardiomyocyte cross-sectional area (CSA) from both the left (LV) and right (RV) ventricles over 18 weeks of TAC. **(A)** Representative images from the LV (top row) and RV (bottom row) stained with wheat germ agglutinin-3. **(B)** Averaged cardiomyocyte CSA over 18 weeks of TAC (*n* = 6 for each point). Complete sham CSA data is presented in **Table 4**. Correlation plots between ventricle weight and CSA in the **(C)** LV and **(D)** RV. ^∗^*p* < 0.01 vs. age-matched sham. ^†^*p* < 0.01 vs. previous time point.

To quantify concentric hypertrophy, cardiomyocyte CSA was assessed in both the LV and RV (**Figure 5**). LV CSA increased progressively over 18 weeks, while RV CSA only increased between 2 and 4 weeks, remaining stable thereafter (**Figure 5B**). The linear increase in LV weight and LV CSA over 18 weeks suggested the rate of hypertrophy was constant and likely (predominantly) concentric (**Figure 5C**). In contrast, the pattern in the RV had two parts. Initially (0–4 weeks), RV CSA and weight increased linearly (**Figure 5D**). Between 4 and 18 weeks, however, RV weight continued to increase while RV CSA plateaued. While this increased weight could be attributed to extracellular proteins or cardiomyocyte hyperplasia, RV fibrosis was static between 4 and 18 weeks (**Figure 2B**) and, adult cardiomyocyte hyperplasia is accepted to be low to negligible. This suggested the weight was coming from an increase in cardiomyocyte cell length (i.e., eccentric hypertrophy).

### Temporal Echocardiography Revealed an Early Period of Stable Cardiac Output Followed by a Secondary Decline and Chamber Dilatation

**FIGURE 6:**
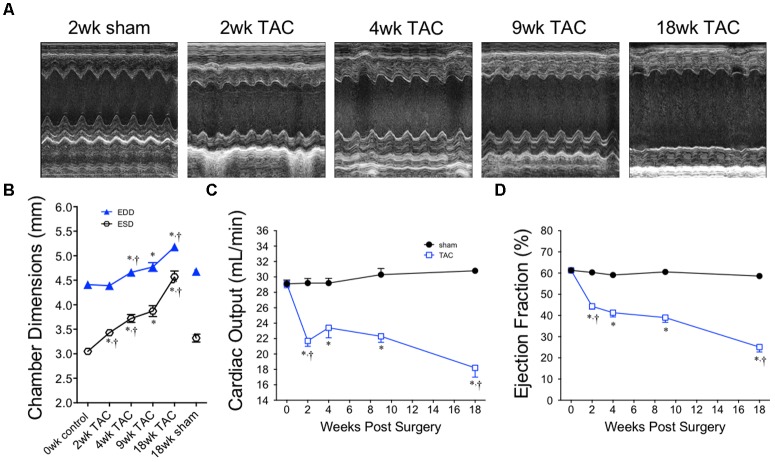
Chamber size and echocardiographic function over 18 weeks of TAC. **(A)** Representative M-mode images acquired from the mid-papillary region of the LV. **(B)** Left ventricular end diastolic (EDD) and end systolic (ESD) dimensions, **(C)** cardiac output and **(D)** ejection fraction demonstrate dilation and a decline in cardiac function, especially between the 9 and 18 week time-points. Heart rate was statistically unchanged between all time points. ^∗^*p* < 0.01 vs. age-matched sham. ^†^*p* < 0.01 vs. previous time point.

Representative M-mode images from the LV of sham and TAC mice are profiled in **Figure 6A**. LV dilation (increased LV end diastolic dimensions) began at 4 weeks and progressed to 18 weeks (**Figure 6B**). Cardiac output decreased by 2 weeks and remained stable until 9 weeks before decreasing further by 18 weeks (**Figure 6C**). Ejection fraction, a common variable used to clinically grade LV function, followed the same pattern as cardiac output with the secondary decline occurring between 9 and 18 weeks (**Figure 6D**). Complete echocardiographic data from sham and TAC mice is summarized in **Table 5**.

**Table 5:** Expanded echocardiographic function.

		TAC			
		
Variable	Pre-surgery (*n* = 8)	2 weeks (*n* = 15)	4 weeks (*n* = 12)	9 weeks (*n* = 17)	18 weeks (*n* = 13)
Dimension_Diastole_ (mm)	4.38 ± 0.04	4.39 ± 0.04	**4.66 ± 0.07**^∗,†^	**4.78 ± 0.07**^∗^	**5.11 ± 0.09**^∗,†^
Dimension_Systole_ (mm)	2.95 ± 0.01	**3.43 ± 0.05**^∗,†^	**3.74 ± 0.08**^∗,†^	**3.86 ± 0.12**^∗^	**4.49 ± 0.13**^∗,†^
Volume_Diastole_ (μL)	86.9 ± 1.8	87.1 ± 1.8	**100.9 ± 3.3**^∗,†^	**107.2 ± 3.8**^∗^	**124.9 ± 4.9**^∗,†^
Volume_Systole_ (μL)	33.5 ± 0.4	**48.6 ± 1.6**^∗,†^	**60.0 ± 3.1**^∗,†^	**65.6 ± 4.9**^∗^	**93.4 ± 5.9**^∗,†^
Stroke Volume (μL)	53.4 ± 1.6	**38.5 ± 1.3**^∗,†^	**40.8 ± 2.3**^∗^	**41.6 ± 1.6**^∗^	**31.5 ± 2.0**^∗,†^
Ejection Fraction (%)	61.3 ± 0.7	**44.3 ± 1.2**^∗,†^	**40.6 ± 2.1**^∗^	**39.9 ± 2.4**^∗^	**26.1 ± 2.2**^∗,†^
Fractional Shortening (%)	32.7 ± 0.5	**21.8 ± 0.8**^∗,†^	**19.9 ± 1.1**^∗^	**19.6 ± 1.3**^∗^	**12.3 ± 1.1**^∗,†^
Cardiac Output (mL/min)	29.1 ± 0.5	**21.9 ± 0.7**^∗,†^	**23.6 ± 1.3**^∗^	**22.8 ± 0.8**^∗^	**22.8 ± 0.8**^∗,†^
Wall Thickness_Diastole_ (mm)	0.88 ± 0.02	**1.15 ± 0.01**^∗,†^	**1.20 ± 0.01**^∗,†^	**1.23 ± 0.01**^∗^	**1.30 ± 0.02**^∗,†^
Heart Rate (bpm)	548 ± 10	570 ± 7	578 ± 13	557 ± 6	575 ± 11^∗^

		**sham**			
		
**Variable**	**Pre-surgery (*n* = 8)**	**2 weeks (*n* = 8)**	**4 weeks (*n* = 8)**	**9weeks (*n* = 9)**	**18 weeks (*n* = 12)**

Dimension_Diastole_ (mm)	4.38 ± 0.04	4.39 ± 0.04	4.44 ± 0.04	4.50 ± 0.07	**4.62 ± 0.04**^#^
Dimension_Systole_ (mm)	2.95 ± 0.01	2.99 ± 0.04	3.05 ± 0.06	3.05 ± 0.08	**3.19 ± 0.06**^#^
Volume_Diastole_ (μL)	86.9 ± 1.8	87.5 ± 1.9	89.5 ± 1.9	92.6 ± 3.7	**98.6 ± 1.8**^#^
Volume_Systole_ (μL)	33.5 ± 0.4	34.8 ± 1.2	36.6 ± 1.7	36.8 ± 2.5	**41.0 ± 1.8**^#^
Stroke Volume (μL)	53.4 ± 1.6	52.7 ± 1.0	52.9 ± 1.2	55.8 ± 1.4	**57.7 ± 0.9**^#^
Ejection Fraction (%)	61.3 ± 0.7	60.3 ± 0.7	59.1 ± 1.4	60.6 ± 1.2	58.7 ± 1.2
Fractional Shortening (%)	32.7 ± 0.5	32.0 ± 0.5	31.2 ± 0.7	32.3 ± 0.8	31.0 ± 0.8
Cardiac Output (mL/min)	29.1 ± 0.5	29.2 ± 0.6	29.2 ± 0.6	30.3 ± 0.8	**30.8 ± 0.3**^#^
Wall Thickness_Diastole_ (mm)	0.88 ± 0.02	0.92 ± 0.03	**0.94 ± 0.03**^#^	**0.97 ± 0.03**^#^	**0.99 ± 0.04**^#^
Heart Rate (bpm)	548 ± 10	554 ± 7	552 ± 11	544 ± 8	535 ± 7


*^∗^*p* < 0.05 vs. age-matched sham mice. ^†^*p* < 0.05 vs. previous time point. ^#^*p* < 0.05 in sham vs. pre-surgery time point. Bolded values draw attention to significance vs. age-matched sham (top) and to significance vs. pre-surgery values (bottom).*

### Pathological Remodeling of the Pulmonary Vasculature Was Progressive With Significant Muscularization of Smaller Vessels

**FIGURE 7:**
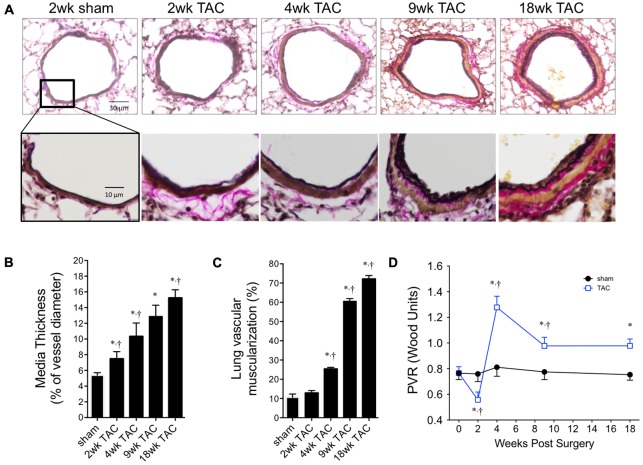
Pulmonary vascular remodeling over 18 weeks of TAC. **(A)** Representative cross-sectional images of whole pulmonary arteries (top panel) and vessel walls (bottom panel). **(B)** Thickness of vascular media was calculated as a percent of external diameter to normalize for varying vessel sizes. **(C)** Percent of muscularized small arterioles (<35 μm) in the lung. Sham values did not change over the 18 weeks study period and are represented as ‘sham’. **(D)** Pulmonary vascular resistance (PVR) was calculated from first principles; Resistance = ΔPressure/Flow. ΔPressure was the gradient between right ventricle systolic pressure and left ventricle end diastolic pressure. Flow was cardiac output. ^∗^*p* < 0.01 vs. age-matched sham. ^†^*p* < 0.05 vs. previous time point.

To confirm the increase in RVP was pathological, we looked for evidence of pulmonary vascular remodeling within the pulmonary circulation by histology (**Figure 7A**). We found increased large-vessel wall thickness by 2 weeks with a neomuscularization of the smaller vessels by 4 weeks; both continuously increased thereafter (**Figures 7B,C**). By 9 weeks, this was associated with an increased total lung weight without evidence of pulmonary edema (i.e., static lung wet/dry weight ratio; **Table 2**). Pulmonary vascular resistance, calculated using the formula PVR = (RVP–LV EDP)/cardiac output), was reduced at 2 weeks, before peaking at 4 weeks and remaining elevated thereafter (**Figure 7D**).

## Discussion

This study is the first to report a longitudinal, biventricular evaluation of cardiac function and morphology in the murine pressure-overload model. Here we mapped the pathophysiological development of type II pulmonary hypertension, pulmonary vascular remodeling and RV dysfunction in TAC for the first time. There were two phases of function, compensatory and decompensatory. In the compensatory phase, both the LV and RV remodeled concentrically in line with increased pressure. During decompensation, the LV continued to hypertrophy concentrically despite declining pressure, while in the RV the data suggested a shift to eccentric hypertrophy. We also demonstrated that no one parameter was sufficient to explain diastolic function with each index (e.g., fibrosis, EDP, tau and dP/dt_min_) following a different pattern over time. And finally, fibrosis did not correlate with diastolic pressure in either ventricle over time, calling into question how fibrosis *per se* is related to elevations in EDP.

### Left Ventricular Perspective

TAC is commonly used to investigate LV hypertrophy as pressure drives proportional concentric cardiac growth (Grossman et al., 1975). The relationship between pressure and hypertrophy over time however, is more complex. In this model, we show early LV hypertrophy was concentric and proportional to pressure (0–4 weeks), but during decompensation (4–18 weeks), concentric hypertrophy persisted despite declining systolic pressures. While the former is well recognized in the literature, the latter (i.e., hypertrophy with declining pressure) is typically only reported at very late time points or in more severe models (Norton et al., 2002; Rothermel et al., 2005; Chen et al., 2012; Mohammed et al., 2012). Thus, pressure-independent hypertrophy is relative and a hallmark of decompensation. Importantly, we confirm the type of hypertrophy during decompensation as concentric. Cardiac hypertrophy is an independent risk factor for cardiovascular morbidity and mortality (Levy et al., 1990), and a primary target of emerging HF therapies (Frey et al., 2004). To our knowledge however, the molecular mechanism(s) by which cardiomyocytes undergo concentric hypertrophy through these two stages have not been directly compared and contrasted. The existence of two phases of concentric hypertrophy is relevant because anti-hypertrophic therapies are often initiated early in a model (e.g., Takimoto et al., 2005). This leaves the impact of therapies in the decompensated stage of cardiac remodeling largely unknown; therapies initiated in this later phase may not have the same outcome as those initiated early on. This is particularly important given the aged clinical population that largely presents after years of hypertrophic remodeling, only as their failing heart begins to precipitate symptoms. As these patients are approaching–or already experiencing–decompensation, comparatively assessing therapies between the compensatory and decompensatory phases is an important future focus of pressure-overload studies.

Both LV hypertrophy and eventual decompensation are ubiquitous following pressure-overload. The variability is in the degree of hypertrophy and the time-to-decompensation. The differences reported between models of pressure-overload are largely contingent on three main features. First, the degree of constriction (gauge of needle) impacts both the amount of hypertrophy and rate of dilation. Smaller gauges [i.e., 24-gauge (Pradhan et al., 2016), 25-gauge (van Nierop et al., 2013)] lead to mild concentric hypertrophy, no early dilation and mild dysfunction. Intermediate gauges [i.e., 27-gauge (Nakamura et al., 2001; Liao et al., 2002; van Nierop et al., 2013)] lead to more substantial short-term hypertrophy, a period of stable or recovered cardiac function, and then dilatation and decompensation within 4 to 9 weeks. Severe constrictions (28-gauge, Rothermel et al., 2005) develop concentric hypertrophy in tandem with rapid dilatation and mortality. Second, animal selection significantly affects the response to pressure-overload. Strain and gender differences contribute to the genetic variation (Barrick et al., 2007, 2009), while different ages and/or weights impact the relative severity of the same-sized constriction (e.g., 27-gauge induces a 20% increase in LV weight by 3 weeks in 6–8 weeks old mice (Rothermel et al., 2005) versus a 50% increase in 14–16 weeks old mice (Kapur et al., 2012)]. Third, and finally, the relative proximity of the band to the heart (i.e., ascending vs. transverse vs. abdominal aortic constriction) also impacts the magnitude of hypertrophy and rate of decompensation (Foster et al., 2017). These data indicate the need for direct intra-study comparisons of hypertrophy and its molecular mechanisms, and that inter-study comparisons be made with these factors in mind. Here, we report early concentric hypertrophy and recovered function (0–4 weeks) before dilatation and decompensation (18 weeks). These data suggest our model was moderate, and thus valuable to investigate the transitional phase between compensatory remodeling and decompensated HF.

Interstitial fibrosis correlates with a stiffening of the LV wall, which impairs ventricular relaxation and contributes (among other things) to increased passive filling pressures (EDP) (Jalil et al., 1989; Weber et al., 1993; Conrad et al., 1995; Yamamoto et al., 2002; Kass et al., 2004; Matsusaka et al., 2006). Increased EDP is part of the diagnosis of diastolic dysfunction (Mandinov et al., 2000; Paulus et al., 2007), thus any cause of increased EDP is a clinically important event. Here we show that over time, an increase in EDP was neither dependent on a change in interstitial fibrosis, nor guaranteed by an increase in interstitial fibrosis. In the LV, fibrosis increased from 7 to 11% between 2 and 18 weeks without commensurate increases in EDP. In the RV, fibrosis increased 5-fold by 2 weeks while EDP remained unchanged. Interestingly, other reports have described disagreement between the onset and severity of fibrosis and the relative diastolic pressures. Wu et al. (2011) reported a threefold increase in LV fibrosis in their model without any change in EDP. Hamdani et al. (2014) reported increased left ventricular interstitial fibrosis in a diabetic model in the absence of changes to EDP. A study by Perrino (2006), reported variable LV interstitial fibrosis with static LVEDP in three different TAC groups. And finally, treating with sildenafil to reduce fibrosis twofold in a TAC model did not alter EDP (Pradhan et al., 2016). These data suggest gross fibrosis does not explain changes in diastolic pressure in TAC. For example, collagen cross-linking is more predictive of LV stiffness and diminished diastolic function than total collagen content, both in experimental models (Badenhorst, 2003; López et al., 2012) and in humans (Ravassa et al., 2017). What remains to be established is a definitive approach to capturing ECM remodeling related to diastolic dysfunction, which will be critical in developing future therapies.

In shams, we report appreciable changes in cardiac structure and function over the 18 week study period. This was especially true in the RV, where between 2 and 9 weeks invasive measures of contractility and relaxation increased by ∼33%. In the same time frame, the change in LV function was <10%. This suggests the healthy RV may reach functional maturity in a different timeframe than the LV. Importantly, evidence of contractile dysfunction in the TAC RV was only significant by 18 weeks because of the increase in RV function in aged-matched shams. This demonstrates the importance of mapping structure and function in shams/controls, particularly in longitudinal studies where early time points may misrepresent the mature phenotype.

### Right Ventricular Perspective

Type-II pulmonary hypertension is the most prevalent form of pulmonary hypertension (Rosenkranz et al., 2016). Reported in over 50% of HF patients (Hoeper et al., 2005, 2009; Lam et al., 2009), its presence increases 3-year mortality by 20–50% (positively correlated with severity of pulmonary hypertension) (Bogaard et al., 2009). While numerous models exist for investi gating the pathophysiology and treatment options for type-I pulmonary hypertension, the literature on experimental models of type-II pulmonary hypertension is comparatively sparse (Stenmark et al., 2009). In type-II pulmonary hypertension, the primary condition is LV dysfunction; increased LV filling pressures (EDP) precede the increase in pulmonary artery pressures (Adusumalli and Mazurek, 2017). This differs etiologi cally from type I pulmonary hypertension, which originates as a primary pathology of the pulmonary vasculature (Simonneau et al., 2009). Here, we corroborate previous findings demonstrating pulmonary hypertension and RV dysfunction are prominent features of the LV pressure-overload model (Chen et al., 2012). We further verified that the onset of pulmonary hypertension was secondary to the increase in EDP, and that pathological remodeling in the pulmonary vasculature was secondary to the spike in RVP. This confirms TAC is a model of type II pulmonary hypertension with the instigating pathology in the LV, which migrates to the RV and associated vasculature. As current therapies for type I pulmonary hypertension (i.e., pulmonary vasodilators) are either ineffective (Naeije and Huez, 2007) or detrimental (Guazzi and Arena, 2010) in patients with HF (type II), there is an urgent need for novel therapies that address this population. TAC presents an ideal model to investigate both the pathophysiology of type II pulmonary hypertension, as well as novel treatments for this prevalent condition.

Remodeling patterns in the LV cannot be applied to the RV; the RV is developmentally (Zaffran, 2004), physiologically (Littlejohns et al., 2014), and molecularly (Fields et al., 1978) distinct from the LV and pumps into a different vascular bed against differing hemodynamic loads. Here we report early concentric hypertrophy between 2 and 4 weeks that occurred in tandem with increased RVP, followed by a secondary phase where RV weight increased without any further increase in cardiomyocyte CSA–suggestive of a shift to eccentric hypertrophy. The lack of concentric hypertrophy was unexpected given sustained pulmonary hypertension. This contrasted the LV where cardiomyocyte CSA increased out to 18 weeks despite declining pressures and suggests the RV does not respond similarly to the stimuli driving continued concentric growth in the late-stage LV. Emerging studies suggest RV hypertrophy may be determinant to mortality; a defining feature of decompensation is a disproportionate increase in RV mass compared to LV mass (Bing et al., 1995; Norton et al., 2002; Brooks et al., 2010; Chen et al., 2012). Generally, cardiac hypertrophy studies are LV-centric (Weeks and McMullen, 2011; Maillet et al., 2013) and more attention should be provided to the RV.

**FIGURE 8:**
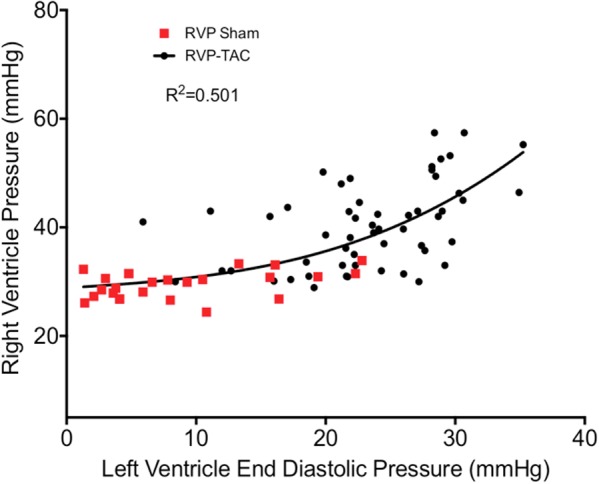
Correlation plot between right ventricle systolic pressure and left ventricular end diastolic pressure in both sham (*n* = 23) and TAC (*n* = 55). The curve of best fit was drawn using a non-linear third order polynomial (cubic) regression.

The RV is, at least in part, hemodynamically dependent on the LV (Santamore and Dell’Italia, 1998). One example of this is in the flow across the lung, which is a function of the pressure gradient between the RV (RVP) and the LV (LVEDP). An increase in LVEDP is thought to cause a reflexive increase in RVP for the maintenance of flow through the pulmonary system (Chen et al., 2012). Our analysis does not show this to be entirely true as a curvilinear relationship is a better fit (**Figure 8**). This may be due to extensive recruitment and distension of pulmonary capillaries to reduce pulmonary vascular resistance in early situations of elevated EDP.

**FIGURE 9:**
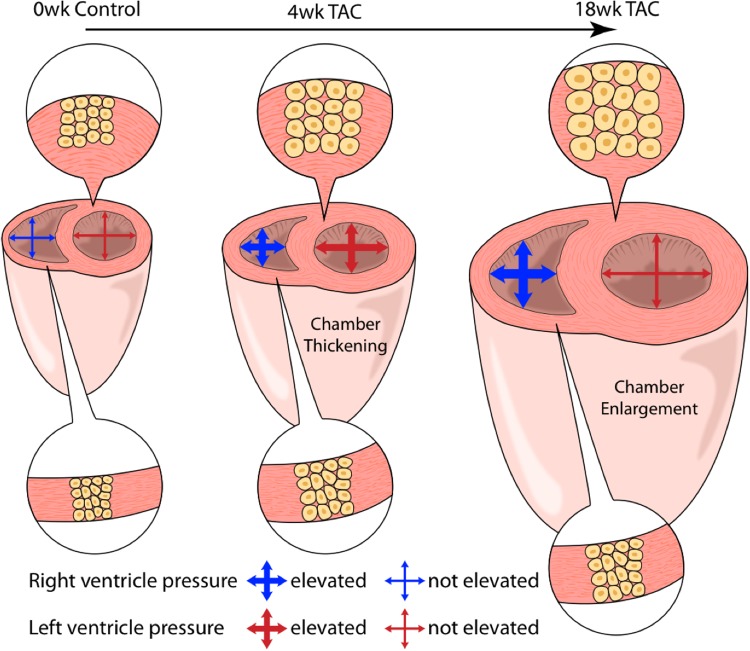
An artistic depiction of the hypertrophic response in both ventricles over 18 weeks of TAC. At 4 weeks, both the right (RV) and left (LV) ventricles present with concentric hypertrophy (thickening of ventricle walls with an increase in cardiomyocyte cross-sectional area (CSA). LV concentric hypertrophy continued out to 18 weeks despite declining systolic pressures. Alternatively, RV cardiomyocyte CSA was static despite sustained systolic pressure-overload. Here, total weight increased in the absence of changes in CSA, suggestive of cardiomyocyte lengthening (eccentric hypertrophy).

Finally, in light of the varying patterns between the LV and RV with regards to pressure and hypertrophy, we introduce an updated depiction of hypertrophy progression in pressure-overload (**Figure 9**).

### Diastolic Dysfunction

The concept of “diastolic dysfunction,” which grew out of clinical observation, is cumbersome to diagnose and a validated gold standard is missing. Indeed, what we define as dysfunction is only in terms of referencing ‘normal’ function, which is dynamic as the cardiovascular system develops and ages. In experimental models, the standard is to measure diastolic function invasively (e.g., EDP, dP/dt_min_, and Tau). This differs from the clinical reliance on indirect surrogate measures (e.g., symptoms, E/A ratio and serum BNP levels). Of concern is whether the surrogate measurements remain in agreement with the fundamental pathophysiology. We found over time, diastolic function was not easily reconciled by any one parameter, suggesting diastolic dysfunction is more a spectrum of impairments than a singular entity.

As components of diastolic function, we interpret EDP as a measure of pressure-stress to relaxation, Tau as the relative time required for relaxation, and dP/dt_min_ as the instantaneous maximum rate-of-relaxation. We show that diastolic parameters were incongruent with each other over time, a finding reported in other pressure-overload studies (Takimoto et al., 2005; Perrino, 2006; Chen et al., 2012). Indeed, each parameter likely has subtle and unique inferential value. If both EDP and Tau are increased, is this the same disease state as increased EDP when Tau is unchanged? As most studies rely on a single parameter to assess diastolic function, this question remains difficult to answer. Caution should be taken when evaluating development of diastolic dysfunction in different etiologies (e.g., channelopathy vs. interstitial fibrosis) as they may not conform to a single diastolic measure. Rather, each measure likely varies in sensitivity to identify dysfunction, or captures a particular physiological nuance of diastolic function.

One discrepancy to highlight is the difference in sensitivity between Tau methods to assess diastolic function. Particularly, Tau Glantz (Tau_G_) detected diastolic impairments by 2 weeks while Tau Weiss (Tau_W_) was unchanged. The discrepancy between Tau measures may exist because Tau_W_ (approximated in isolated hearts) assumes an independence of Tau from volume loading and changes in EDP (Weiss et al., 1976). Tau_G_, however, is an updated method derived from observations in intact dog hearts, and accounts for the impact of variable loading conditions (i.e., changing diastolic pressures) (Raff and Glantz, 1981). Given the chronically elevated EDP in our (and most) pressure overload model(s), Tau_G_ may be the more sensitive parameter. Yet, it remains uncertain at this time which is the superior measure that avoids type-I/II statistical errors and, thus, until more physiological studies are performed, both measures should be used with a clear knowledge as to their underlying assumptions.

Additionally, it was unexpected that in sham mice, while systolic function remained stable, the thresholds for diastolic dysfunction were met (EDP, dP/dt_min_, and Tau_G_ decreased from pre-surgical values). This is consistent with human data where diastolic function is more fragile with aging than systolic function (Fischer et al., 2003). These steady declines suggest the “thresholds” used to identify diastolic dysfunction may need to be adjusted in each study based on reference values relative to aging.

Finally, parameters for grading diastolic function in the LV were also impaired in the RV following TAC. However, RV diastolic dysfunction remains largely undefined, both clinically and in basic research, and gold standard indicies of RV diastolic function remain to be determined in a patient population (Axell et al., 2015).

### Congestion in Pressure-Overload

HF, either clinically or experimentally, is not defined by the presence or absence of pulmonary edema. Indeed, congestion is neither always expected in HF nor always due to pulmonary edema. In fact, pulmonary edema is rare in patients with HF (Mahdyoon et al., 1989; Tarvasmäki et al., 2014; Melenovsky et al., 2015), particularly when compared to the reference values in age-matched individuals without HF (Kataoka and Matsuno, 2008). This is sometimes despite the presence of hemodynamic congestion (this was part of the motivation to shift terminology from congestive HF to HF with preserved or reduced ejection fraction). Consistent with HF patients, a feature of TAC is congestion without pulmonary edema (Chen et al., 2012; Foster et al., 2017). This is not to say that pulmonary edema never occurs in experimental models of HF. Both in our study and in others, pulmonary edema is observed along with rapid decompensation instead of reaching pre-determined time-points (Inoko et al., 1994; Kihara and Sasayama, 1997; Siri et al., 1997; Chung et al., 1998; van Nierop et al., 2013). Thus, when present, pulmonary edema in rodent models may be more indicative of impending mortality than part of the pathophysiology of morbidity.

### Limitations

While we characterize cardiac function and morphology across multiple time points, we do so in young healthy male mice when the clinical HF population is variably aged and both sexes. Indeed, the necessary next step is to apply this ‘mapping’ approach using aged male and female subjects. Future pre-clinical models might also benefit from an assessment of frequently used clinical variables such as blood biomarkers (i.e., BNP; Doust et al., 2005), Doppler echo, or frailty indexes (Singh et al., 2014) to further improve the alignment between experimental models and the clinical population. As well, isolated cardiomyocyte measurements of length and width would provide insight into eccentric hypertrophy.

### Future Outlook

The goal of HF research is to translate findings to the clinical population to improve survival and quality of life. As too few drugs make the transition from bench to bedside (Mak et al., 2014; Fernández-Avilés et al., 2018), there is a need to improve the success of translational research. Several reports have emphasized that the current designs of preclinical rodent studies are not fundamentally rigorous enough (Kloner, 2013; Ovize et al., 2013; Vander Heide and Steenbergen, 2013). This demands an improved understanding of the basic pathophysiology of the models used to investigate preclinical therapies (Houser et al., 2012). A bi-ventricular temporal assessment incorporating multiple indices of diastolic function addresses this in part, adding to our understanding of pressure-overload HF and specifically the time course of pathology in the RV.

## Author Contributions

MP, JH, KB, and JS conceptualized the study, designed the experiments, drafted the manuscript, critically revised the manuscript for intellectual content, and gave the final approval for the article. MP, NR, and JH conducted the experiments. All authors analyzed and/or interpreted the experimental results and read and gave permission to the final draft of the manuscript.

## Conflict of Interest Statement

The authors declare that the research was conducted in the absence of any commercial or financial relationships that could be construed as a potential conflict of interest.

## Abbreviations

BW: body weight
CSA: cross-sectional area
EDP: End Diastolic Pressure
HF: heart failure
HW: heart weight
LV: left ventricle
LVP: left ventricular pressure
RV: right ventricle
RVP: right ventricular pressure
TAC: transverse aortic constriction
TL: tibia length

## References

[B1] Adusumalli S. Mazurek J. A. (2017). Pulmonary hypertension due to left ventricular cardiomyopathy: is it the result or cause of disease progression? 14 507–513. 10.1007/s11897-017-03682 PMC5846484 29063280

[B2] Allwood M. A. Kinobe R. T. Ballantyne L. Romanova N. Melo L. G. Ward C. A. (2014). Heme oxygenase-1 overexpression exacerbates heart failure with aging and pressure overload but is protective against isoproterenol-induced cardiomyopathy in mice. 23 231–237. 10.1016/j.carpath.2014.03.007 24813593

[B3] Axell R. G. Hoole S. P. Hampton-Till J. White P. A. (2015). RV diastolic dysfunction: time to re-evaluate its importance in heart failure. 20 363–373. 10.1007/s10741-015-9472-0 25633340

[B4] Badenhorst D. (2003). Cross-linking influences the impact of quantitative changes in myocardial collagen on cardiac stiffness and remodelling in hypertension in rats. 57 632–641. 10.1016/S0008-6363(02)00733-2 12618225

[B5] Barrick C. J. Dong A. Waikel R. Corn D. Yang F. Threadgill D. W. (2009). Parent-of-origin effects on cardiac response to pressure overload in mice. 297 H1003–H1009. 10.1152/ajpheart.00896.2008 19561308 PMC2755989

[B6] Barrick C. J. Rojas M. Schoonhoven R. Smyth S. S. Threadgill D. W. (2007). Cardiac response to pressure overload in 129S1/SvImJ and C57BL/6J mice: temporal- and background-dependent development of concentric left ventricular hypertrophy. 292 H2119–H2130. 10.1152/ajpheart.00816.2006 17172276

[B7] Benjamin E. J. Blaha M. J. Chiuve S. E. Cushman M. Das S. R. Deo R. (2017). Heart disease and stroke statistics-2017 update: a report from the American Heart Association. 135:e646. 10.1161/CIR.0000000000000485 28122885 PMC5408160

[B8] Bing O. H. Brooks W. W. Robinson K. G. Slawsky M. T. Hayes J. A. Litwin S. E. (1995). The spontaneously hypertensive rat as a model of the transition from compensated left ventricular hypertrophy to failure. 27 383–396. 10.1016/S0022-2828(08)80035-1 7760360

[B9] Bogaard H. J. Abe K. Vonk Noordegraaf A. Voelkel N. F. (2009). The right ventricle under pressure: cellular and molecular mechanisms of right-heart failure in pulmonary hypertension. 135 794–804. 10.1378/chest.08-0492 19265089

[B10] Brooks W. W. Shen S. S. Conrad C. H. Goldstein R. H. Bing O. H. L. (2010). Transition from compensated hypertrophy to systolic heart failure in the spontaneously hypertensive rat: structure, function, and transcript analysis. 95 84–92. 10.1016/j.ygeno.2009.12.002 20006699

[B11] Chen Y. Guo H. Xu D. Xu X. Wang H. Hu X. (2012). Left ventricular failure produces profound lung remodeling and pulmonary hypertension in mice: heart failure causes severe lung disease. 59 1170–1178. 10.1161/HYPERTENSIONAHA.111.186072 22508832 PMC3402091

[B12] Chung E. S. Perlini S. Aurigemma G. P. Fenton R. A. Dobson J. G. Meyer T. E. (1998). Effects of chronic adenosine uptake blockade on adrenergic responsiveness and left ventricular chamber function in pressure overload hypertrophy in the rat. 16 1813–1822. 10.1097/00004872-199816120-00015 9869016

[B13] Conrad C. H. Brooks W. W. Hayes J. A. Sen S. Robinson K. G. Bing O. H. (1995). Myocardial fibrosis and stiffness with hypertrophy and heart failure in the spontaneously hypertensive rat. 91 161–170. 10.1161/01.CIR.91.1.161 7805198

[B14] Doust J. A. Pietrzak E. Dobson A. Glasziou P. (2005). How well does B-type natriuretic peptide predict death and cardiac events in patients with heart failure: systematic review. 330:625. 10.1136/bmj.330.7492.625 15774989 PMC554905

[B15] Fernández-Avilés F. Sanz-Ruiz R. Climent A. M. Badimon L. Bolli R. Charron D. (2018). Global overview of the transnational alliance for regenerative therapies in cardiovascular syndromes (TACTICS) recommendations: a comprehensive series of challenges and priorities of cardiovascular regenerative medicine. 122 199–201. 10.1161/CIRCRESAHA.117.312099 29348246

[B16] Ferreira R. G. Worthington A. Huang C.-C. Aranki S. F. Muehlschlegel J. D. (2015). Sex differences in the prevalence of diastolic dysfunction in cardiac surgical patients. 30 238–245. 10.1111/jocs.12506 25571945 PMC4573536

[B17] Fields J. Z. Roeske W. R. Morkin E. Yamamura H. I. (1978). Cardiac muscarinic cholinergic receptors. Biochemical identification and characterization. 253 3251–3258. 641068

[B18] Fischer M. Baessler A. Hense H. W. Hengstenberg C. Muscholl M. Holmer S. (2003). Prevalence of left ventricular diastolic dysfunction in the community. Results from a Doppler echocardiographic-based survey of a population sample. 24 320–328. 10.1016/S0195-668X(02)00428-1 12581679

[B19] Foster A. J. Platt M. J. Huber J. S. Eadie A. L. Arkell A. M. Romanova N. (2017). Central-acting therapeutics alleviate respiratory weakness caused by heart failure-induced ventilatory overdrive. 9:eaag1303. 10.1126/scitranslmed.aag1303 28515334

[B20] Frey N. Katus H. A. Olson E. N. Hill J. A. (2004). Hypertrophy of the heart: a new therapeutic target? 109 1580–1589. 10.1161/01.CIR.0000120390.68287.BB 15066961

[B21] Ghio S. Gavazzi A. Campana C. Inserra C. Klersy C. Sebastiani R. (2001). Independent and additive prognostic value of right ventricular systolic function and pulmonary artery pressure in patients with chronic heart failure. 37 183–188. 10.1016/S0735-1097(00)01102-5 11153735

[B22] Grossman W. Jones D. McLaurin L. P. (1975). Wall stress and patterns of hypertrophy in the human left ventricle. 56 56–64. 10.1172/JCI108079 124746 PMC436555

[B23] Guazzi M. Arena R. (2010). Pulmonary hypertension with left-sided heart disease. 7 648–659. 10.1038/nrcardio.2010.144 20924360

[B24] Hamdani N. Hervent A.-S. Vandekerckhove L. Matheeussen V. Demolder M. Baerts L. (2014). Left ventricular diastolic dysfunction and myocardial stiffness in diabetic mice is attenuated by inhibition of dipeptidyl peptidase 4. 104 423–431. 10.1093/cvr/cvu223 25341892

[B25] Heidenreich P. A. Trogdon J. G. Khavjou O. A. Butler J. Dracup K. Ezekowitz M. D. (2011). Forecasting the future of cardiovascular disease in the United States: a policy statement from the American Heart Associatio. 123 933–944. 10.1161/CIR.0b013e31820a55f5 21262990

[B26] Hoeper M. M. Barberà J. A. Channick R. N. Hassoun P. M. Lang I. M. Manes A. (2009). Diagnosis, assessment, and treatment of non-pulmonary arterial hypertension pulmonary hypertension. 54 S85–S96. 10.1016/j.jacc.2009.04.008 19555862

[B27] Hoeper M. M. Markevych I. Spiekerkoetter E. Welte T. Niedermeyer J. (2005). Goal-oriented treatment and combination therapy for pulmonary arterial hypertension. 26 858–863. 10.1183/09031936.05.00075305 16264047

[B28] Hogg K. Swedberg K. McMurray J. (2004). Heart failure with preserved left ventricular systolic function; epidemiology, clinical characteristics, and prognosis. 43 317–327. 10.1016/j.jacc.2003.07.046 15013109

[B29] Houser S. R. Margulies K. B. Murphy A. M. Spinale F. G. Francis G. S. Prabhu S. D. (2012). Animal models of heart failure: a scientific statement from the American Heart Association. 111 131–150. 10.1161/RES.0b013e3182582523 22595296

[B30] Inoko M. Kihara Y. Morii I. Fujiwara H. Sasayama S. (1994). Transition from compensatory hypertrophy to dilated, failing left ventricles in Dahl salt-sensitive rats. 267 H2471–H2482. 10.1152/ajpheart.1994.267.6.H2471 7810745

[B31] Jalil J. E. Doering C. W. Janicki J. S. Pick R. Shroff S. G. Weber K. T. (1989). Fibrillar collagen and myocardial stiffness in the intact hypertrophied rat left ventricle. 64 1041–1050. 10.1161/01.RES.64.6.1041 2524288

[B32] Kapur N. K. Wilson S. Yunis A. A. Qiao X. Mackey E. Paruchuri V. (2012). Reduced endoglin activity limits cardiac fibrosis and improves survival in heart failure. 125 2728–2738. 10.1161/CIRCULATIONAHA.111.080002 22592898 PMC4774533

[B33] Karaye K. M. Habib A. G. Mohammed S. Rabiu M. Shehu M. N. (2010). Assessment of right ventricular systolic function using tricuspid annular-plane systolic excursion in Nigerians with systemic hypertension. 21 186–190. 10.5830/CVJA-2010-031 20838715 PMC3721899

[B34] Kass D. A. Bronzwaer J. G. F. Paulus W. J. (2004). What mechanisms underlie diastolic dysfunction in heart failure? 94 1533–1542. 10.1161/01.RES.0000129254.25507.d6 15217918

[B35] Kataoka H. Matsuno O. (2008). Age-related pulmonary crackles (rales) in asymptomatic cardiovascular patients. 6 239–245. 10.1370/afm.834 18474887 PMC2384982

[B36] Kihara Y. Sasayama S. (1997). Transition from compensatory hypertrophy to dilated failing left ventricle in Dahl-Iwai salt-sensitive rats. 10 78S–82S. 10.1016/S0895-7061(97)00080-0 9160786

[B37] Kloner R. A. (2013). Current state of clinical translation of cardioprotective agents for acute myocardial infarction. 113 451–463. 10.1161/CIRCRESAHA.112.300627 23908332

[B38] Klotz S. Hay I. Zhang G. Maurer M. Wang J. Burkhoff D. (2006). Development of heart failure in chronic hypertensive Dahl rats: focus on heart failure with preserved ejection fraction. 47 901–911. 10.1161/01.HYP.0000215579.81408.8e 16585423

[B39] Lam C. S. P. Roger V. L. Rodeheffer R. J. Borlaug B. A. Enders F. T. Redfield M. M. (2009). Pulmonary hypertension in heart failure with preserved ejection fraction. 53 1119–1126. 10.1016/j.jacc.2008.11.051 19324256 PMC2736110

[B40] Levy D. Garrison R. J. Savage D. D. Kannel W. B. Castelli W. P. (1990). Prognostic implications of echocardiographically determined left ventricular mass in the Framingham Heart Study. 322 1561–1566. 10.1056/NEJM199005313222203 2139921

[B41] Liao Y. Ishikura F. Beppu S. Asakura M. Takashima S. Asanuma H. (2002). Echocardiographic assessment of LV hypertrophy and function in aortic-banded mice: necropsy validation. 282 H1703–H1708. 10.1152/ajpheart.00238.2001 11959634

[B42] Littlejohns B. Heesom K. Angelini G. D. Suleiman M.-S. (2014). The effect of disease on human cardiac protein expression profiles in paired samples from right and left ventricles. 11 1–14. 10.1186/1559-0275-11-34 25249829 PMC4158351

[B43] López B. Querejeta R. González A. Larman M. Díez J. (2012). Collagen cross-linking but not collagen amount associates with elevated filling pressures in hypertensive patients with stage C heart failure: potential role of lysyl oxidase. 60 677–683. 10.1161/HYPERTENSIONAHA.112.196113 22824984

[B44] Mahdyoon H. Klein R. Eyler W. Lakier J. B. Chakko S. C. Gheorghiade M. (1989). Radiographic pulmonary congestion in end-stage congestive heart failure. 63 625–627. 10.1016/0002-9149(89)90912-0 2919567

[B45] Maillet M. van Berlo J. H. Molkentin J. D. (2013). Molecular basis of physiological heart growth: fundamental concepts and new players. 14 38–48. 10.1038/nrm3495 23258295 PMC4416212

[B46] Mak I. W. Evaniew N. Ghert M. (2014). Lost in translation: animal models and clinical trials in cancer treatment. 6 114–118. 24489990 PMC3902221

[B47] Mandinov L. Eberli F. R. Seiler C. Hess O. M. (2000). Diastolic heart failure. 45 813–825. 10.1016/S0008-6363(99)00399-5 10728407

[B48] Matsusaka H. Ide T. Matsushima S. Ikeuchi M. Kubota T. Sunagawa K. (2006). Targeted deletion of matrix metalloproteinase 2 ameliorates myocardial remodeling in mice with chronic pressure overload. 47 711–717. 10.1161/01.HYP.0000208840.30778.00 16505197

[B49] Melenovsky V. Andersen M. J. Andress K. Reddy Y. N. Borlaug B. A. (2015). Lung congestion in chronic heart failure: haemodynamic, clinical, and prognostic implications. 17 1161–1171. 10.1002/ejhf.417 26467180

[B50] Meyer P. Filippatos G. S. Ahmed M. I. Iskandrian A. E. Bittner V. Perry G. J. (2010). Effects of right ventricular ejection fraction on outcomes in chronic systolic heart failure. 121 252–258. 10.1161/CIRCULATIONAHA.109.887570 20048206 PMC2877272

[B51] Mirsky I. Pfeffer J. M. Pfeffer M. A. Braunwald E. (1983). The contractile state as the major determinant in the evolution of left ventricular dysfunction in the spontaneously hypertensive rat. 53 767–778. 10.1161/01.RES.53.6.767 6640863

[B52] Mohammed S. F. Storlie J. R. Oehler E. A. Bowen L. A. Korinek J. Lam C. S. P. (2012). Variable phenotype in murine transverse aortic constriction. 21 188–198. 10.1016/j.carpath.2011.05.002 21764606 PMC3412352

[B53] Naeije R. Huez S. (2007). Expert opinion on available options treating pulmonary arterial hypertension. 8 2247–2265. 10.1517/14656566.8.14.2247 17927481

[B54] Nakamura A. Rokosh D. G. Paccanaro M. Yee R. R. Simpson P. C. Grossman W. (2001). LV systolic performance improves with development of hypertrophy after transverse aortic constriction in mice. 281 H1104–H1112. 10.1152/ajpheart.2001.281.3.H1104 11514276

[B55] Norton G. R. Woodiwiss A. J. Gaasch W. H. Mela T. Chung E. S. Aurigemma G. P. (2002). Heart failure in pressure overload hypertrophy: the relative roles of ventricular remodeling and myocardial dysfunction. 39 664–671. 10.1016/S0735-1097(01)01792-2 11849866

[B56] Ovize M. Thibault H. Przyklenk K. (2013). Myocardial conditioning: opportunities for clinical translation. 113 439–450. 10.1161/CIRCRESAHA.113.300764 23908331

[B57] Paulus W. J. Tschöpe C. Sanderson J. E. Rusconi C. Flachskampf F. A. Rademakers F. E. (2007). How to diagnose diastolic heart failure: a consensus statement on the diagnosis of heart failure with normal left ventricular ejection fraction by the Heart Failure and Echocardiography Associations of the European Society of Cardiology. 28 2539–2550. 10.1093/eurheartj/ehm037 17428822

[B58] Perrino C. (2006). Intermittent pressure overload triggers hypertrophy-independent cardiac dysfunction and vascular rarefaction. 116 1547–1560. 10.1172/JCI25397 16741575 PMC1464895

[B59] Platt M. J. Huber J. S. Brunt K. R. Simpson J. A. (2017). Pulmonary flow as an improved method for determining cardiac output in mice after myocardial infarction. 30 612.e–623.e. 10.1016/j.echo.2017.02.008 28528655

[B60] Pradhan K. Sydykov A. Tian X. Mamazhakypov A. Neupane B. Luitel H. (2016). Soluble guanylate cyclase stimulator riociguat and phosphodiesterase 5 inhibitor sildenafil ameliorate pulmonary hypertension due to left heart disease in mice. 216 85–91. 10.1016/j.ijcard.2016.04.098 27140341

[B61] Raff G. L. Glantz S. A. (1981). Volume loading slows left ventricular isovolumic relaxation rate. Evidence of load-dependent relaxation in the intact dog heart. 48 813–824. 10.1161/01.RES.48.6.813 7226443

[B62] Ravassa S. López B. Querejeta R. Echegaray K. San José G. Moreno M. U. (2017). Phenotyping of myocardial fibrosis in hypertensive patients with heart failure. Influence on clinical outcome. 35 853–861. 10.1097/HJH.0000000000001258 28253222

[B63] Rockman H. A. Ross R. S. Harris A. N. Knowlton K. U. Steinhelper M. E. Field L. J. (1991). Segregation of atrial-specific and inducible expression of an atrial natriuretic factor transgene in an *in vivo* murine model of cardiac hypertrophy. 88 8277–8281. 10.1073/pnas.88.18.8277 1832775 PMC52490

[B64] Rosenkranz S. Gibbs J. S. R. Wachter R. De Marco T. Vonk Noordegraaf A. Vachiéry J.-L. (2016). Left ventricular heart failure and pulmonary hypertension. 37 942–954. 10.1093/eurheartj/ehv512 26508169 PMC4800173

[B65] Rothermel B. A. Berenji K. Tannous P. Kutschke W. Dey A. Nolan B. (2005). Differential activation of stress-response signaling in load-induced cardiac hypertrophy and failure. 23 18–27. 10.1152/physiolgenomics.00061.2005 16033866 PMC4118287

[B66] Santamore W. P. Dell’Italia L. J. (1998). Ventricular interdependence: significant left ventricular contributions to right ventricular systolic function. 40 289–308. 10.1016/S0033-0620(98)80049-2 9449956

[B67] Sheikh A. Q. Lighthouse J. K. Greif D. M. (2014). Recapitulation of developing artery muscularization in pulmonary hypertension. 6 809–817. 10.1016/j.celrep.2014.01.042 24582963 PMC4015349

[B68] Simonneau G. Robbins I. M. Beghetti M. Channick R. N. Delcroix M. Denton C. P. (2009). Updated clinical classification of pulmonary hypertension. 54 S43–S54. 10.1016/j.jacc.2009.04.012 19555858

[B69] Singh M. Stewart R. White H. (2014). Importance of frailty in patients with cardiovascular disease. 35 1726–1731. 10.1093/eurheartj/ehu197 24864078 PMC4565652

[B70] Siri F. M. Jelicks L. A. Leinwand L. A. Gardin J. M. (1997). Gated magnetic resonance imaging of normal and hypertrophied murine hearts. 272 H2394–H2402. 10.1152/ajpheart.1997.272.5.H2394 9176310

[B71] Stenmark K. R. Meyrick B. Galiè N. Mooi W. J. McMurtry I. F. (2009). Animal models of pulmonary arterial hypertension: the hope for etiological discovery and pharmacological cure. 297 L1013–L1032. 10.1152/ajplung.00217.2009 19748998

[B72] Takimoto E. Champion H. C. Li M. Belardi D. Ren S. (2005). Chronic inhibition of cyclic GMP phosphodiesterase 5A prevents and reverses cardiac hypertrophy. 11 214–222. 10.1038/nm1175 15665834

[B73] Tarvasmäki T. Harjola V.-P. Nieminen M. S. Siirilä-Waris K. Tolonen J. Tolppanen H. (2014). Acute heart failure with and without concomitant acute coronary syndromes: patient characteristics, management, and survival. 20 723–730. 10.1016/j.cardfail.2014.07.008 25079300

[B74] van Nierop B. J. van Assen H. C. van Deel E. D. Niesen L. B. P. Duncker D. J. Strijkers G. J. (2013). Phenotyping of left and right ventricular function in mouse models of compensated hypertrophy and heart failure with cardiac MRI. 8:e55424. 10.1371/journal.pone.0055424 23383329 PMC3562232

[B75] Vander Heide R. S. Steenbergen C. (2013). Cardioprotection and myocardial reperfusion: pitfalls to clinical application. 113 464–477. 10.1161/CIRCRESAHA.113.300765 23908333 PMC3824252

[B76] Wan S.-H. Vogel M. W. Chen H. H. (2014). Pre-clinical diastolic dysfunction. 63 407–416. 10.1016/j.jacc.2013.10.063 24291270 PMC3934927

[B77] Weber K. T. Brilla C. G. Janicki J. S. (1993). Myocardial fibrosis: functional significance and regulatory factors. 27 341–348. 10.1093/cvr/27.3.341 8490934

[B78] Weeks K. L. McMullen J. R. (2011). The athlete’s heart vs. the failing heart: can signaling explain the two distinct outcomes? 26 97–105. 10.1152/physiol.00043.2010 21487028

[B79] Weiss J. L. Frederiksen J. W. Weisfeldt M. L. (1976). Hemodynamic determinants of the time-course of fall in canine left ventricular pressure. 58 751–760. 10.1172/JCI108522 956400 PMC333234

[B80] Wu X. Simpson J. Hong J. H. Kim K.-H. Thavarajah N. K. Backx P. H. (2011). MEK-ERK pathway modulation ameliorates disease phenotypes in a mouse model of Noonan syndrome associated with the *Raf1^L613V^* mutation. 121 1009–1025. 10.1172/JCI44929 21339642 PMC3049402

[B81] Yamamoto K. Masuyama T. Sakata Y. Nishikawa N. Mano T. Yoshida J. (2002). Myocardial stiffness is determined by ventricular fibrosis, but not by compensatory or excessive hypertrophy in hypertensive heart. 55 76–82. 10.1016/S0008-6363(02)00341-3 12062710

[B82] Zaffran S. (2004). Right ventricular myocardium derives from the anterior heart field. 95 261–268. 10.1161/01.RES.0000136815.73623.BE 15217909

[B83] Zakeri R. Mohammed S. F. (2015). Epidemiology of right ventricular dysfunction in heart failure with preserved ejection fraction. 12 295–301. 10.1007/s11897-015-0267-3 26338372

